# A first step towards understanding thermomechanical behavior of the Nb-Cr system through interatomic potential development and molecular dynamics simulations

**DOI:** 10.1038/s41598-024-64920-w

**Published:** 2024-06-22

**Authors:** Lucas A. Heaton, Adib J. Samin

**Affiliations:** Department of Engineering Physics, Air Force Institute of Technology, Wright-Patterson Air Force Base, Dayton, 45433 USA

**Keywords:** Materials science, Structural materials, Mechanical properties, Metals and alloys

## Abstract

Utilizing a preliminary interatomic potential, this work represents an initial exploration into the thermomechanical behavior of NbCr solid solutions. Specifically, it examines the effect of different amounts of Cr solute, for which information in the literature is limited. The employed interatomic potential was developed according to the embedded atom model (EAM), and was trained on data derived from density functional theory calculations. While the potential demonstrated reasonable accuracy and predictive power when tested, various results highlight deficiencies and encourage further development and training. Mechanical strength, heat capacities, thermal expansion coefficients, and thermal conductivities were found to decrease with Cr content. Elastic coefficients, too, were observed to be strongly dependent on Cr composition. The Pugh embrittlement criterion was not satisfied for any of the compositions and temperatures explored. Gibbs free energy calculations performed on C14, C15, and C36 NbCr$_{2}$ allotropes predicted the C36 structure to be the most thermodynamically favorable across all investigated temperatures and it was found that C36 becomes increasingly more stable relative to the other two phases with increased pressure. The inability of this work to accurately capture the stability of the different Laves phases is most likely due to the shortcomings in the developed potential.

## Introduction

**Figure 1:**
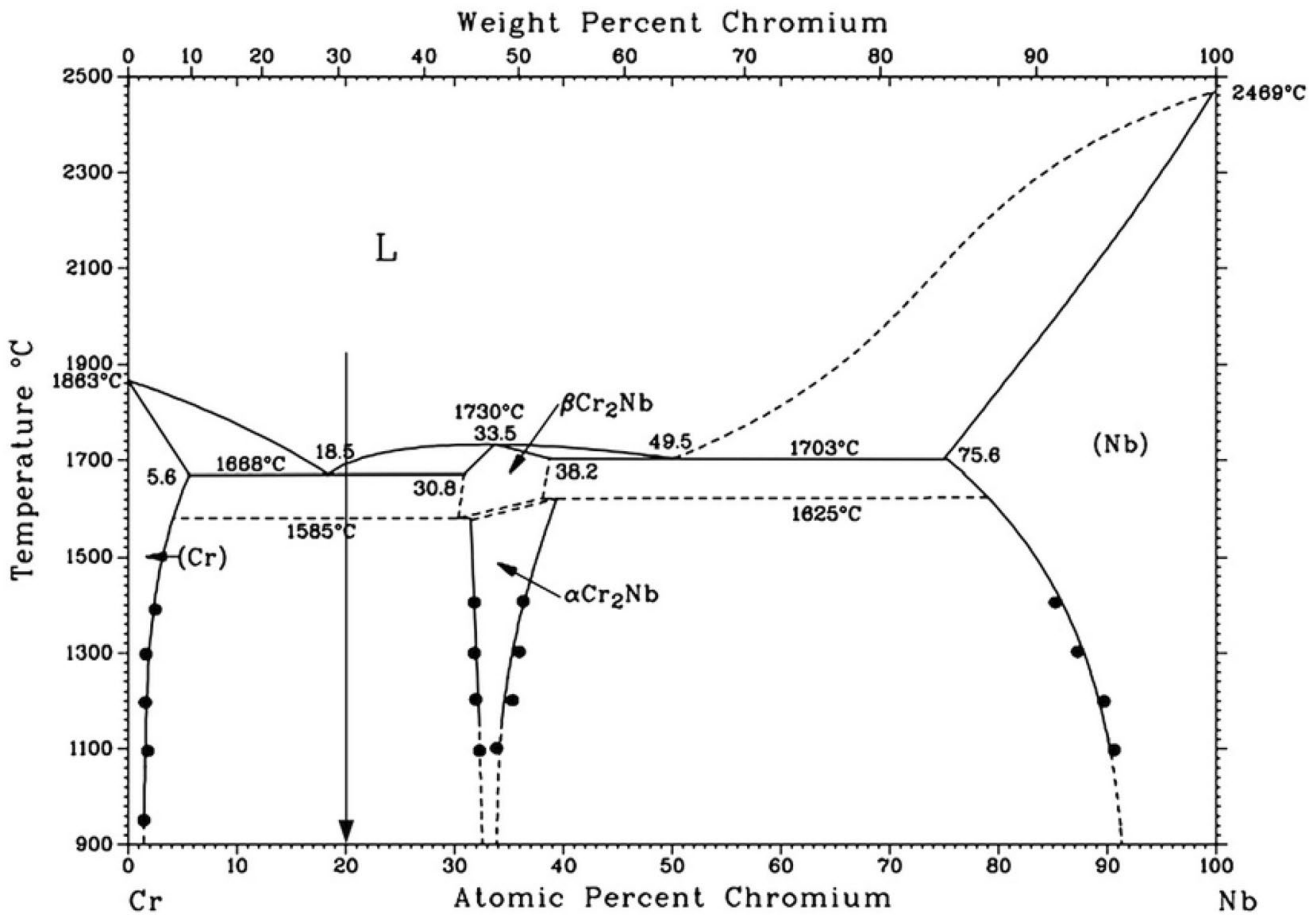
Nb-Cr phase diagram from Li et al^32^ showing the C15 phase, $\alpha$Cr$_{2}$Nb, and the C14 phase, $\beta$Cr$_{2}$Nb.

Largely payable to their refractory nature, Nb-Cr compounds are of significant interest in a variety of high-temperature applications, as well as in radiation shielding studies^1–9^. Nb-Cr alloys, for example, have been considered as potential cladding materials in nuclear reactors^10^. Additionally, with higher melting temperatures and lower densities than prominent nickel-based alloys^11^, Nb and Cr represent a potential enhancement in material utility for a variety of aerospace uses^12,13^. While these attractive applications have motivated an extensive body of literature on the Nb-Cr system, there are still relationships which have yet to be explored and questions in need of addressing. Primarily, there exists a lack of information regarding Nb-Cr alloys and the impact of chromium solutes on the macroscopic properties of body-centered cubic (bcc) niobium-based solid solutions. This gap is particularly significant given the relatively high solubility of Cr in Nb, compared to the solubility of Nb in Cr (see Fig. 1). A recent study by Butler et al^11^ found that the CrNb alloy consisted of a NbCr$_{2}$ Laves phase matrix and Nb-rich bcc precipitates, consistent with known phase diagrams and the one obtained from CALPHAD. The work also reported that CrNb exhibited the highest strength up to 1200$^\circ$C, but was the most brittle at 25$^\circ$C and 1000$^\circ$C upon compression testing. Additionally, Butler et al found that ductility in a metastable Cr$_{49.9}$Nb$_{49.5}$ alloy was improved by the addition of Ta and Ti metals, but this came at the cost of reduced compressive strength.

While there is a dearth of information on the behavior of solid solution Nb-Cr alloys in the literature, the mechanical properties of NbCr$_{2}$-based alloys have been studied at length^1,3,4,14–25^. As NbCr$_{2}$ exhibits high thermal stability and exceptional corrosion and creep resistance due to its highly-complex close-packed crystal structure^22^, material applications span a wide range. C15 NbCr$_{2}$ crystallizes in the face-centered cubic Bravais lattice in the Fd$\overline{3}$m space group and has been observed over a wide range of temperatures. C14 adopts a hexagonal unit cell in the P6$_{3}$/mmc space group and has been observed between approximately 1800 K and its melting temperature around 2100 K^2,26,27^. Studying the C15 structure, Liu et al^4^ found that certain doping elements had a pronounced effect on the mechanical properties of NbCr$_{2}$. Namely, the study reported a dramatic increase in NbCr$_{2}$ tensile and shear strength with the introduction of Hf or Si. The authors concluded that the enhancement stemmed from achieving isotropic behavior in NbCr$_{2}$ and reported a negative correlation between lattice distortion caused by the introduction of ternary elements and material strength. Huang et al^1^ reached a similar conclusion in 2022, writing that, depending on the impact to the bulk crystal structure, the extent to which NbCr$_{2}$’s properties may be optimized through alloying is limited. Both studies emphasized the importance of preserving the Laves structure as much as possible, as it was responsible for NbCr$_{2}$’s high-temperature strength. The effect of alloying elements on NbCr$_{2}$ properties has also been examined under plastic deformation. Takasugi’s stress-strain analyses of C15 NbCr$_{2}$^21,23^ found that the stress-strain response of the material is not impacted by small perturbations in the concentration of additional elements. Kazantzis et al^22^ also carried out an experimental stress-strain analyses of C15 NbCr$_{2}$ and reported the nucleation of $\frac {1}{2}\mathrm {<}$110$\mathrm {>}$ dislocations as the dominant deformation mechanism. The work also concluded that dislocation motion occurred at high temperature due to a relaxation in the otherwise tightly-packed Laves-phase crystal structure, which created large Peierls forces for dislocation motion. In another experimental study utilizing spark plasma sintering, Gao et al^19^ observed that crack propagation in Laves NbCr$_{2}$ proceeded in a zigzag pattern, improving fracture toughness. Additional studies on Laves NbCr$_{2}$ have included investigations of thermal properties. Samanta et al, in two different works^24,28^, carried out experiments to determine the thermal expansion coefficients and heat capacities of C15 NbCr$_{2}$ as a function of temperature. Additionally, Jiang et al^27^ calculated heat capacities and Gibbs energies.

In addition to the knowledge gap on Nb-Cr alloys, there exists lingering disagreement among scientists regarding the phase equilibria of the Laves-phase NbCr$_{2}$ compounds. While various thermodynamic evaluations of the NbCr$_{2}$ Laves phases have mostly agreed^2,26,27^, recent literature has debated the stability of the hexagonal C14 NbCr$_{2}$ allotrope, with some suggesting that the cubic C15 NbCr$_{2}$ structure existed up until the melting temperature^29–31^. Aufrecht et al^29^ triggered the debate, determining that the C15 to C14 phase transition around 1800 K did not occur for sufficiently pure NbCr$_{2}$ alloys and stated that previous experimental studies supporting the existence of an equilibrium C14 state did not adhere to rigorous purity and cleanliness standards. However, in 2023, Hajra et al^25^ claimed to resolve the matter, predicting a C15 to C14 transition at 1971 K via DFT and a C36 (another Laves structure) to C14 transition below 1273 K via experimental methods. In this work, we attempt to address some of these issues and gain insight into the effects of Cr solutes in Nb by developing structure-property relationships based on molecular dynamics simulations performed using an interatomic potential we developed for the Nb-Cr system.

## Results

In the ensuing paragraphs, we present calculations for several thermal and mechanical properties of Laves-phase NbCr$_{2}$ and three Nb-Cr bcc alloys. To help qualify our findings and give context to our results, we begin with an evaluation of the interatomic potential trained for this work. Frequent comparisons to literature also aid the discussion.

### Interatomic potential

**Figure 2:**
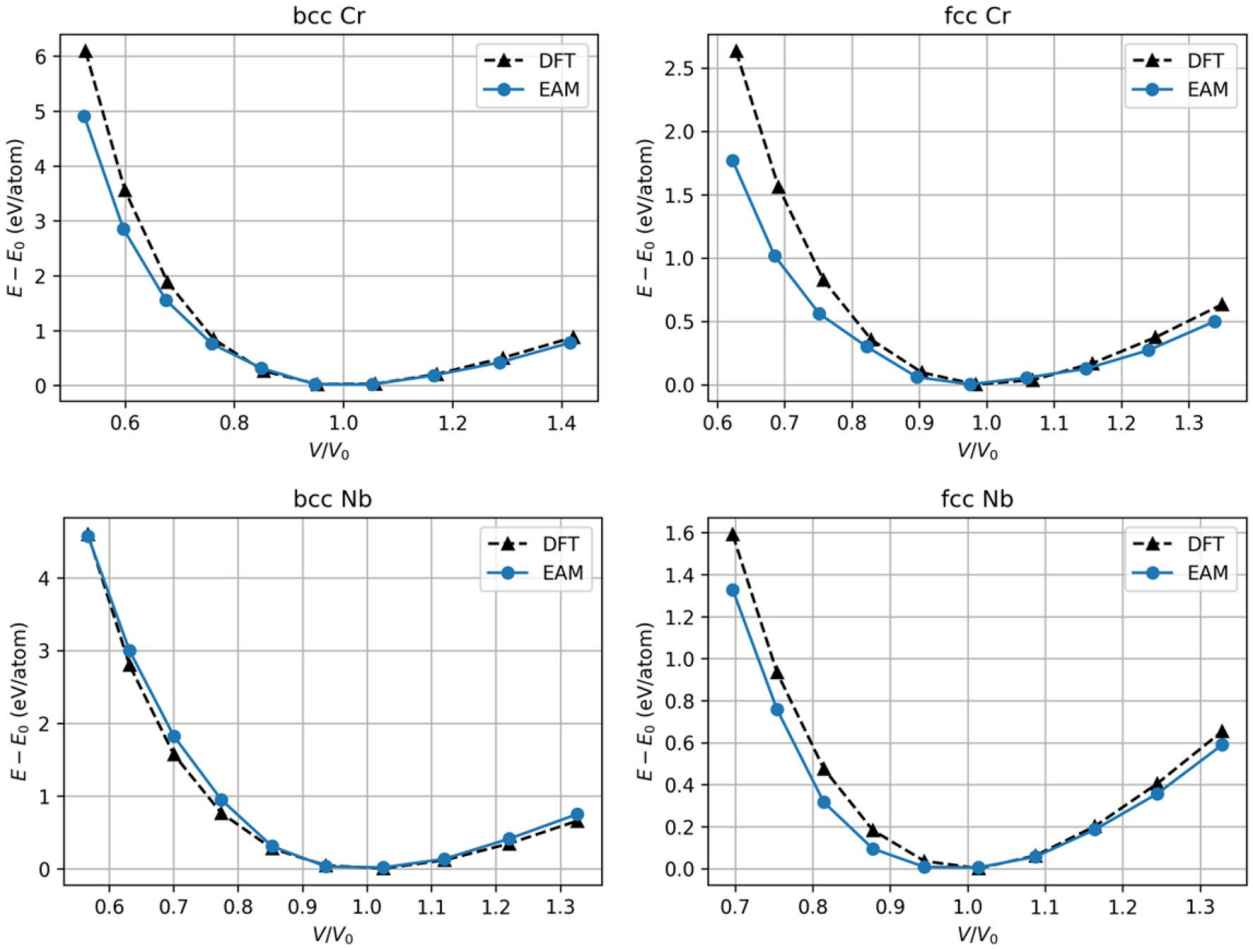
Comparison of EAM and DFT-predicted equations of state (training structures).

**Figure 3:**
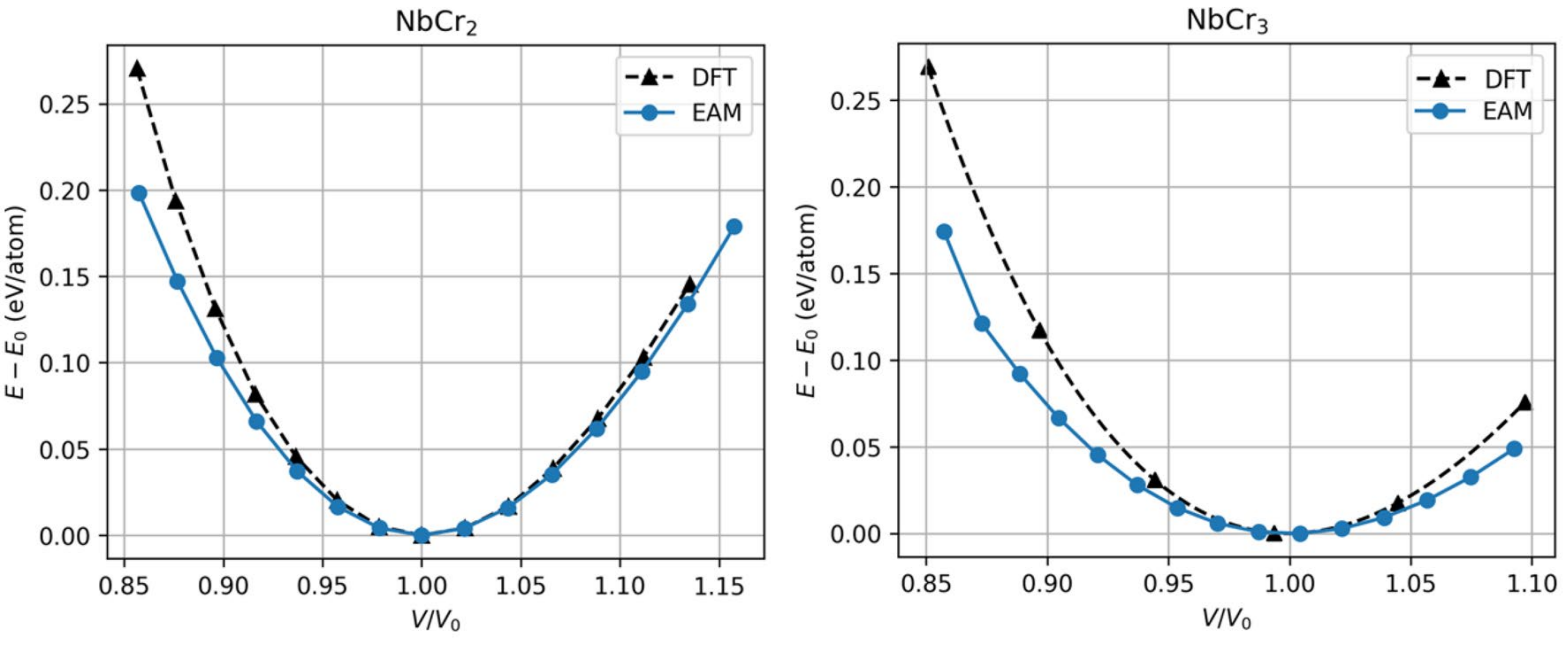
Predictive Power of EAM Potential: Equations of State.

**Figure 4:**
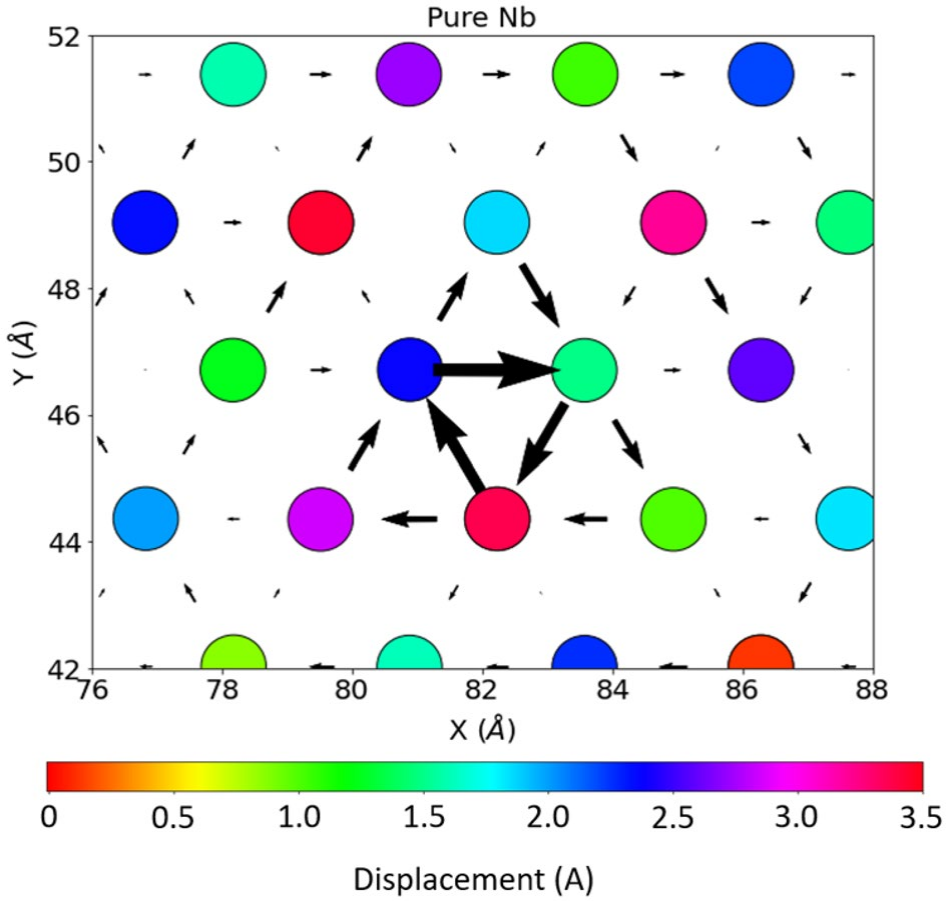
Differential displacement map of a $\frac {1}{2}\mathrm {<}$111$\mathrm {>}$ screw dislocation core at 0 K in pure Nb. The three bold arrows pointing clockwise in the center reflect a non-degenerate, compact dislocation core.

As outlined in Section 4.2, an interatomic potential based on the embedded-atom model (EAM) was developed to describe the Nb-Cr interactions for this work. First, an EAM interatomic potential describing Nb-Nb interactions was trained using the lattice constant, equations of state, the cohesive energy and elastic coefficients for both bcc and fcc phases. A similar approach was taken for describing the Cr-Cr interactions. Finally, the Nb-Cr cross interaction was described using Johnson’s^33^ method based on the two single element EAM potentials without any additional training. All fitted parameters are shown in Table 1. For Cr, the dependence on electron density suggested in Equation 12 is ignored and only one $F_{3}$ parameter is used. A comparison of the EAM-calculated values and those calculated by Density Functional Theory (DFT) for various physical properties of each training structure is shown in Table 2. The corresponding equations of state are plotted in Fig. 2. To test the predictive power of the EAM potential and the ability of the cross-term formulation introduced by Johnson^33^ to capture the *a*-*b* interactions, lattice constants, elastic coefficients, cohesive energies, and equations of state were calculated for two NbCr$_{x}$ intermetallics and compared with DFT data. These predictions and the comparison to DFT are shown in Table 3 and Fig. 3. Finally, as this work reports on various mechanical properties of Nb-rich solid solutions, which are governed by the core of $\frac {1}{2}\mathrm {<}$111$\mathrm {>}$ screw dislocations^34^, Fig. 4 includes a differential displacement map of the core structure for a $\frac {1}{2}\mathrm {<}$111$\mathrm {>}$ screw dislocation in pure Nb.

**Table 1:**
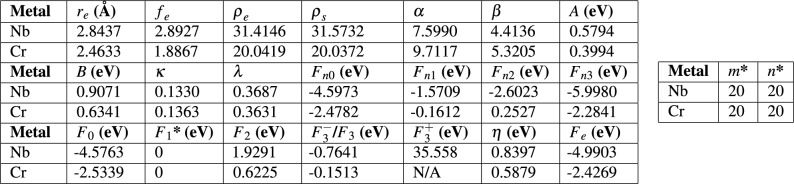
Fitted EAM parameters. * identifies parameters that were held constant during the fitting process.

As shown in Table 2 and Fig. 2, the potential demonstrates reasonable agreement with the DFT based values it was trained on for both bcc and fcc phases. While there is a significant degree of disagreement in the elastic coefficients predicted by the two methods, particularly for $C_{11}$ and $C_{12}$ in Cr, recent literature highlights the difficulty in accurately predicting these quantities^35^. The EAM potential is observed to predict equations of state in each material to an acceptable degree of accuracy, as shown in Fig. 2.

The predictive power of the EAM potential is highlighted in Table 3 and Fig. 3. The two intermetallics chosen were C15 NbCr$_{2}$ and a cubic NbCr$_{3}$ structure. By visual inspection of Fig. 3, it can seen that the EAM potential closely predicts energy as a function of volume near $V=V_{0}$ but begins to deviate from the DFT predictions at volumes far from $V_{0}$. This behavior, along with the comparison in Table 3, demonstrates acceptable predictive power. Furthermore, the discrepancies in the intermetallic predictions exhibit a similar degree of congruity with the discrepancies in the training data calculations (Table 2).

In a final display of our potential’s suitability for the present work, Fig. 4 reveals the core structure of a $\frac {1}{2}\mathrm {<}$111$\mathrm {>}$ screw dislocation predicted by the employed potential. The three larger arrows in the center, surrounded by six smaller arrows in the clockwise direction reflects a non-degenerate, compact core, consistent with numerous works in the literature^36–41^.

**Table 2:**
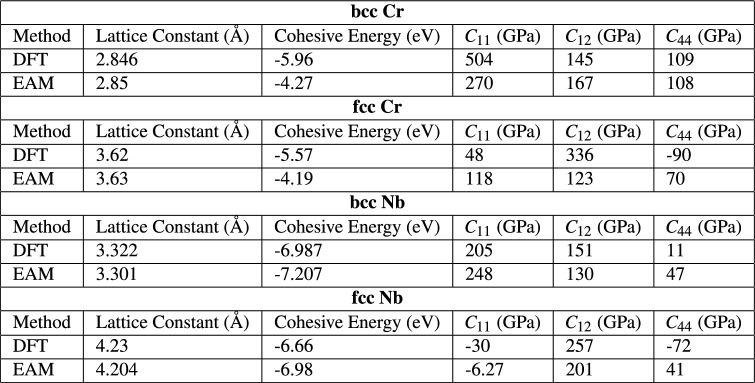
EAM-calculated physical properties of structures used for training, in comparison with DFT values.

**Table 3:**
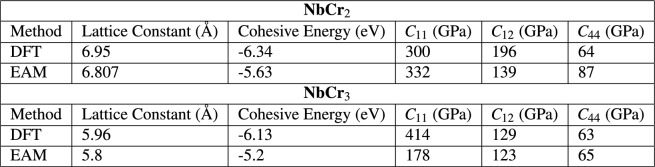
Predictive Power of EAM Potential: Physical Properties.

### Stress-strain response

**Figure 5:**
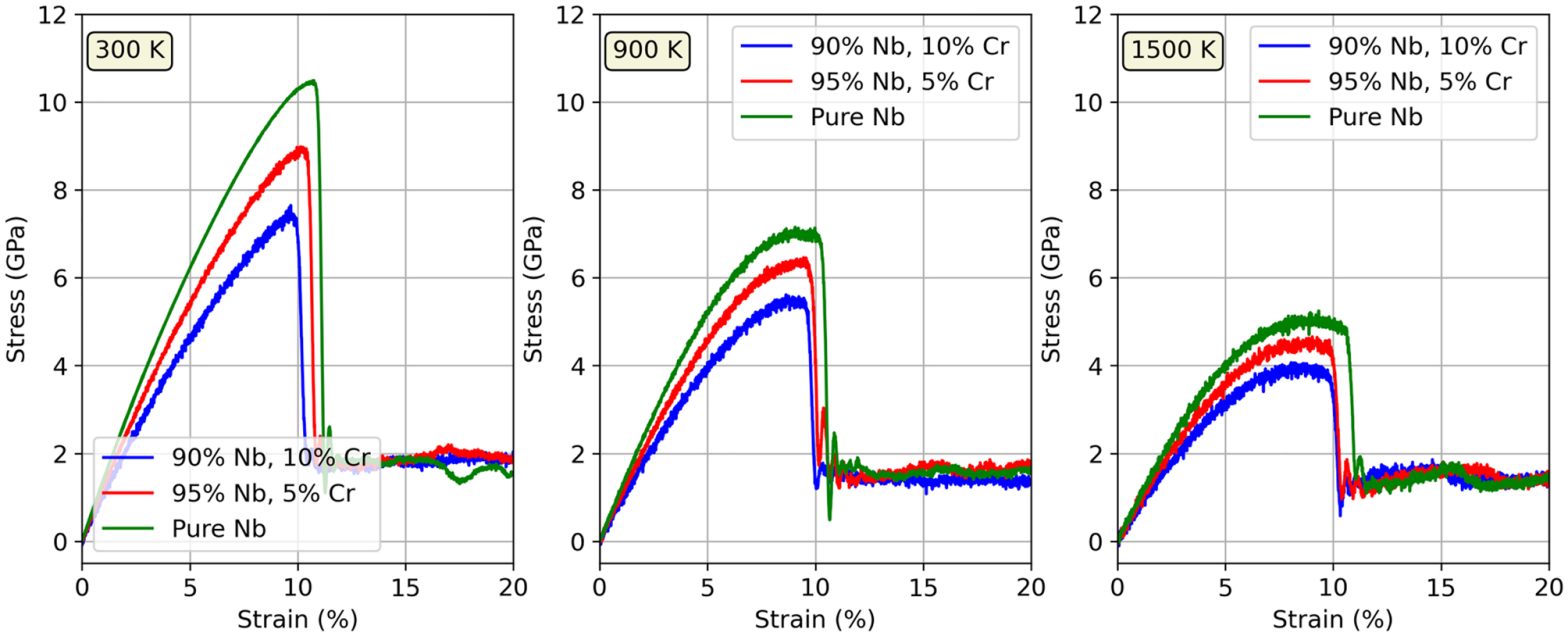
Stress-strain responses in three Nb-Cr alloys. All curves correspond to an engineering strain rate of 0.001/fs. The temperature is shown in the top left of each plot.

Stress-strain curves of three single crystal Nb-Cr alloys were generated by uniaxially stretching each sample in the [100] crystal direction and recording the corresponding component of the stress tensor. The results are captured in Fig. 5. At a glance, a reduction in the peak height of each stress-strain curve is observed from 300 K to 1500 K, mirroring the reduction in material strength observed with increasing temperature as reported in literature^21–23^. Additionally, it can be seen that pure Nb exhibits the highest strength and demonstrates the highest ductility of all alloys over the wide range of temperatures examined. This is consistent with Jiang et al^42^, which reported a decrease in ductility in W-Cr alloys with increases in Cr content up to the dilute limit at 0 K. However, as high strength is empirically linked to low ductility^11,21–23^, the behavior shown in our stress strain curves is unexpected and may be attributed to inadequacies in our employed potential. In exploring strain rate dependence (see Fig. 1 in the Supplementary Information), we found that ultimate tensile strength increased with strain rate, which is consistent with multiple sources in the literature^43–47^. Stress-strain curves for C14 and C15 NbCr$_{2}$ may be found in Fig. 2 of the Supplementary Information. We found that both Laves phases exhibited higher strength than the alloys, but also demonstrated high ductility. This is at odds with the literature^1,3,4,14^, which typically characterizes these phases as brittle due to the absence of sufficient slip systems for easy dislocation motion. We attribute this behavior to the interatomic potential. We also observe that - for both structures - material strength is nearly constant from 300 K to 1500 K (as compared to the Nb-based solid solutions), supporting the idea of high thermal stability in Laves phases.

**Figure 6:**
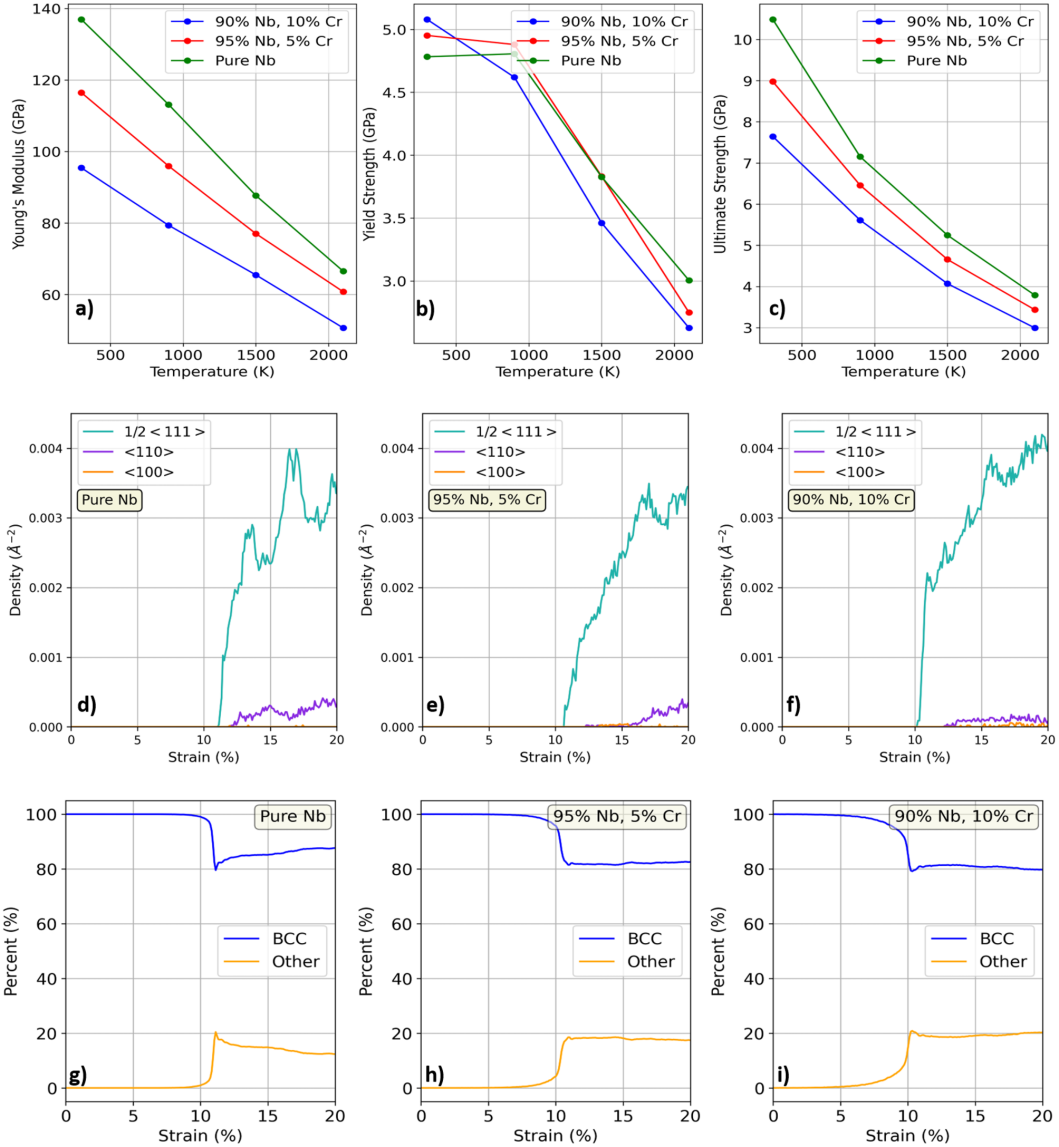
Summary of mechanical behavior in Nb-Cr alloys. All data points were calculated from the curves shown in Fig. 5 (i.e. tensile strain along [100] crystal direction at a rate of 0.001/fs). Figures d–i correspond to a temperature of 300 K. **a–c)** Young’s modulus, yield strength, and ultimate tensile strength. **d–f)** Dislocation densities as a function of strain. The inverse area units correspond to dislocation densities measured in terms of dislocation length per unit volume. **g–i)** Phase transformations as a function of strain.

To better illustrate the effect of increased Cr content on the mechanical properties of Nb-Cr alloys, we plot Young’s modulus, yield strength, and ultimate tensile strength, as well as dislocation densities, and phase transformations in each alloy in Fig. 6. All results are shown for a strain rate of 0.001/fs. As the stress-strain curves are continuous and lack apparent yielding behavior, yield strengths were determined by using the 0.2% offset method. We observe that, in line with Takasugi’s works^21,23^ and Kazantzis et al^22^, Young’s modulus, yield strength, and ultimate tensile strength all decrease as temperature increases. Furthermore, it’s important to note that these parameters also decrease with Cr content. We also note that the anomalously high yield strength in the 10 at% Cr alloy at 300 K is consistent with pronounced size factors (mismatch in the atomic radii between the solute atoms and the solvent atoms) as defined by solid solution strengthening, which predicts a compressive strain field near the solute that attracts dislocations. At higher temperatures, the yield strength in the 10 at% Cr alloy decreases and becomes less than that of either the pure Nb or the 5 at% Cr alloy. This behavior supports the idea of a reduced impact from the size factor on solid solution strengthening at high temperatures^48,49^. The absence of solid solution strengthening in the ultimate tensile strength plot, however, is not consistent with our intuition and is likely a shortcoming of the interatomic potential and requires further investigation. Additionally, we show an increase in both strength and ductility with higher Cr content. This is at odds with several studies which observed higher strength accompanied by a decrease in ductility^11,21–23^.

Dislocation densities as a function of strain are shown in Fig. 6d–f. It is observed that increased Cr content leads to earlier onset of dislocations, consistent with a lower yield strength. While this behavior aligns with the slightly lower ductility observed with increased Cr content, it does not agree with predictions from solid solution strengthening and may be attributed to the deficiency of the devised potential. Additionally, for all three alloys, dislocations with Burgers vectors along $\frac {1}{2}\mathrm {<}$111$\mathrm {>}$, which are known to be important in bcc structures, dominate the population of dislocations and begin to nucleate around 10% strain. This onset of dislocation nucleation is consistent with Fig. 5, which highlights entry into the plastic deformation stage for each material around the same, 10% strain. Fig. 6g–i show the corresponding phase transformations of each material under uniaxial strain at 300 K. It can be seen that the onset of phase transformation in the Nb-Cr alloys occurs for lower strains with increasing amounts of Cr. Phase transformations were calculated using OVITO’s^50^ polyhedral template matching (PTM) algorithm, which assigns atoms to known crystal structures based on nearest-neighbor configurations. The algorithm is capable of identifying a wide range of structures (sc, bcc, fcc, hcp, icosahedral, cubic diamond, hexagonal diamond, and graphene) and therefore is a robust tool for capturing the local structural environment inside a solid.

### Crack propagation

**Figure 7:**
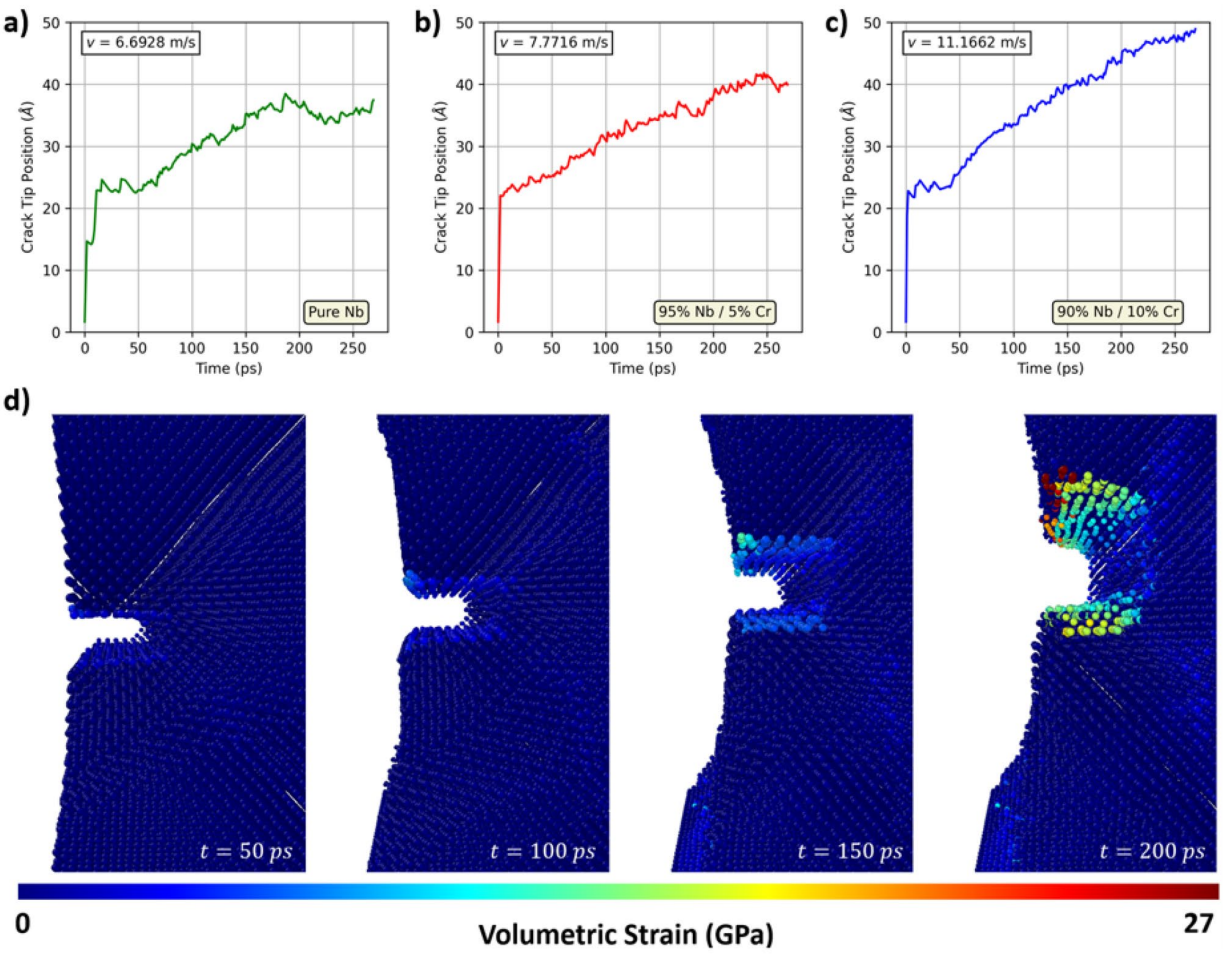
Crack propagation in Nb. **a–c**) Crack tip position as a function of time. **d**) Visualization of crack propagation in pure Nb. All atoms are color-coded according to volumetric strain.

Related to fracture toughness, crack growth rates are a useful metric to consider for describing a material’s durability and resistance to fracture. In Fig. 7, it can be observed that increasing amounts of Cr act to increase the crack growth rate in various Nb-Cr alloys and reduce fracture toughness. This trend is consistent with the stress-strain behavior seen in Fig. 5. The approximate velocities of the crack tip in each material are shown in the upper left hand corner of each plot. The methodology for this study, including how Fig. 7 was generated, is included in Sect.4.3. Figure 7d shows the evolution of the crack in pure Nb as a function of time. The crack tip is blunted as early as t = 50 ps.

### Elastic coefficients

Elastic coefficients are crucial for understanding a material’s behavior in the elastic deformation regime. Below, we report the effect of increasing amounts of Cr on the elastic coefficients of a Nb-rich alloy by plotting the temperature dependence of the C$_{11}$, C$_{12}$, and C$_{44}$ components of the elastic coefficient matrix. In examining Fig. 9, our results demonstrate that increasing amounts of Cr act to reduce C$_{11}$ across all temperatures, increase C$_{44}$, but have an inconclusive effect on C$_{12}$. As C$_{11}$ represents the hardness in a cubic system, the increasing amounts of Cr are found to reduce material hardness in Nb-based solid solutions. This is in qualitative agreement with the work of Jiang et al^51^ which reported improved hardness in W-Mo alloys with larger amounts of Mo. This behavior is also consistent with Fig. 5, as Young’s modulus is observed to be lower in the alloys than in the pure metal. Similar to the effect of Cr solutes on yield strength in Fig. 6b, we also observe a solid solution strengthening effect in examining the temperature dependence of C$_{44}$. At low temperatures, C$_{44}$ is highest in the 10 at% alloy and lowest in the pure Nb with values ranging by approximately 13 GPa in magnitude. However, at 1500 K, C$_{44}$ falls within 53 ± 3 GPa for all three materials, supporting a diminished size factor impact on strengthening. Similarly, in comparing the bulk modulus, shear modulus, and Poisson’s ratio with a linear interpolation (Fig. 10), we observe that the bulk moduli and Poisson’s ratio exhibit linear behavior. This finding aligns with the screw dislocation solid solution strengthening models proposed by Suzuki^52^, as well as Maresca and Curtin^53^, which say that the concentration dependence of strengthening in dilute alloys (<10% solute) is generally linear in *x*, except at very low temperatures. Poisson’s ratio was calculated via Equation 6.

**Figure 8:**
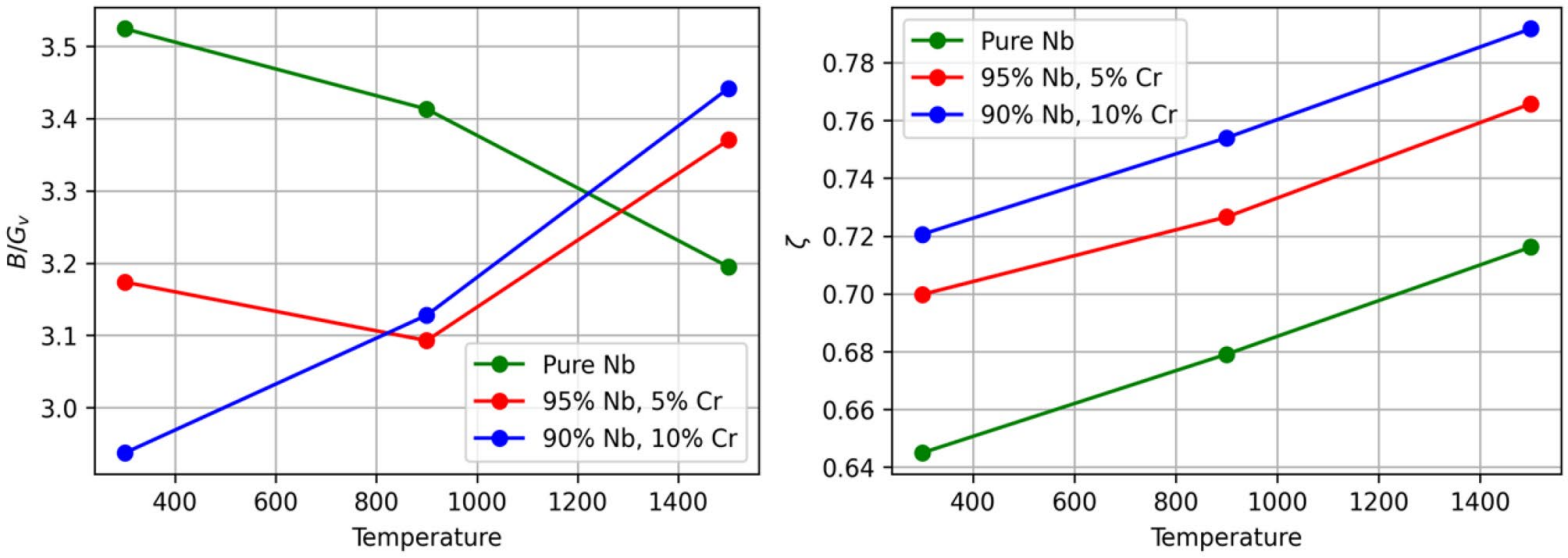
Pugh’s criterion as a function of temperature (left) and the Kleinman parameter as a function of temperature (right).

In terms of general behavior, Pugh’s criterion (Fig. 8) predicts that all three alloys are ductile ($B/G_{v}$ > 1.75) over the entire temperature range, indicating an absence of embrittlement for this composition and temperature range. Additionally, all three alloys satisfy the well-known Born-Huang elastic stability criteria^18^ for cubic structures over the entire temperature range. These criteria are outlined in equations 1, 1, and 2. The bulk modulus, *B*, and shear modulus, $G_{v}$, were calculated according to equations 3 and 4. Finally, using Kleinman’s parameter^54^ to describe the internal strain of each alloy, we predict that bond stretching ($\zeta =1$), as opposed to bond bending ($\zeta =0$), is dominant in each material, becoming more prominent with increasing Cr content. This is a reasonable conclusion, given that Cr has a slightly lower atomic radius compared to niobium (128 pm vs 146 pm). Kleinman’s parameter is given by Equation 5. Elastic coefficients were also calculated for C15 NbCr$_{2}$ and may be found in Table 4. The Laves phase material was also found to exhibit bond stretching as opposed to bond bending. Furthermore, C15 elasticity is observed to obey the Born-Huang criteria. All behaviors are consistent with the findings of Long et al^18^.

1 $$\begin{aligned}{} & {} C_{11}-\left| C_{12}\right| > 0 \end{aligned}$$

2 $$\begin{aligned}{} & {} C_{11}+2C_{12} > 0 \end{aligned}$$

3 $$\begin{aligned}{} & {} C_{44} > 0 \end{aligned}$$

4 $$\begin{aligned}{} & {} B=(C_{11}+2C_{12})/3 \end{aligned}$$

5 $$\begin{aligned}{} & {} G_{v}=(C_{11}-C_{12}+3C_{44})/5 \end{aligned}$$

6 $$\begin{aligned}{} & {} \zeta =\frac{C_{11}+8C_{12}}{7C_{11}+2C_{12}} \end{aligned}$$

7 $$\begin{aligned}{} & {} \nu =\frac{1}{2}-\frac{E}{6B} \end{aligned}$$

**Table 4:** Various measures of elasticity in C15 NbCr$_{2}$ as a function of temperature. With the exception of Poisson’s ratio, all quantities are in units of GPa.

**Quantity**	**0 K**	**300 K**	**900 K**	**1500 K**
C$_{11}$	350.53	311.46	278.72	250.84
C$_{12}$	148.41	141.74	133.94	125.18
C$_{44}$	91.48	88.26	82.42	75.29
*B*	215.78	198.31	182.20	167.07
$G_{\nu }$	95.31	86.90	78.41	70.31
$\nu$	0.3075	0.3088	0.3155	0.3118

**Figure 9:**
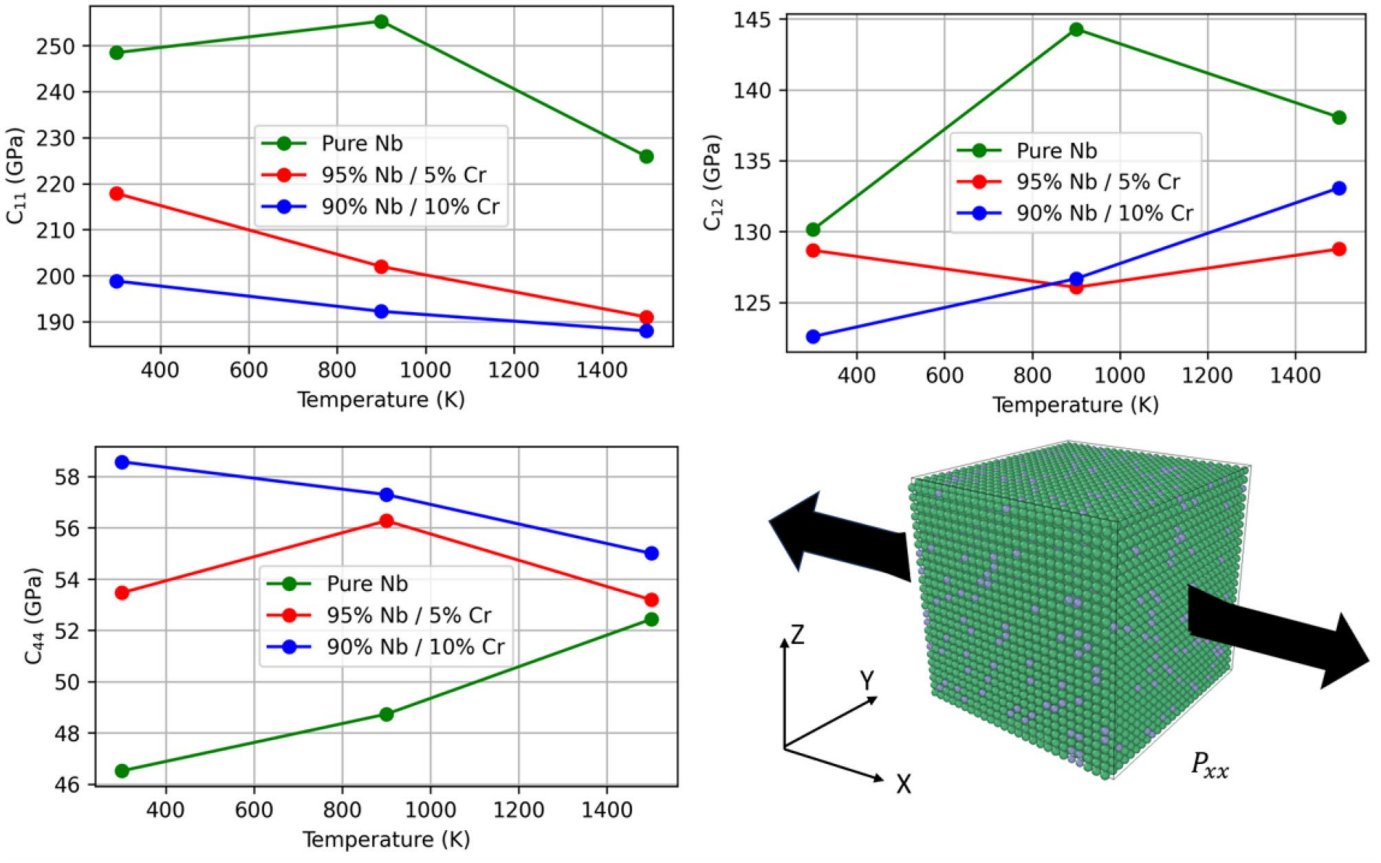
Elastic coefficients for three Nb-Cr alloys. The bottom right figure shows activation of the P$_{xx}$ stress component in the 95% Nb, 5% Cr alloy.

**Figure 10:**
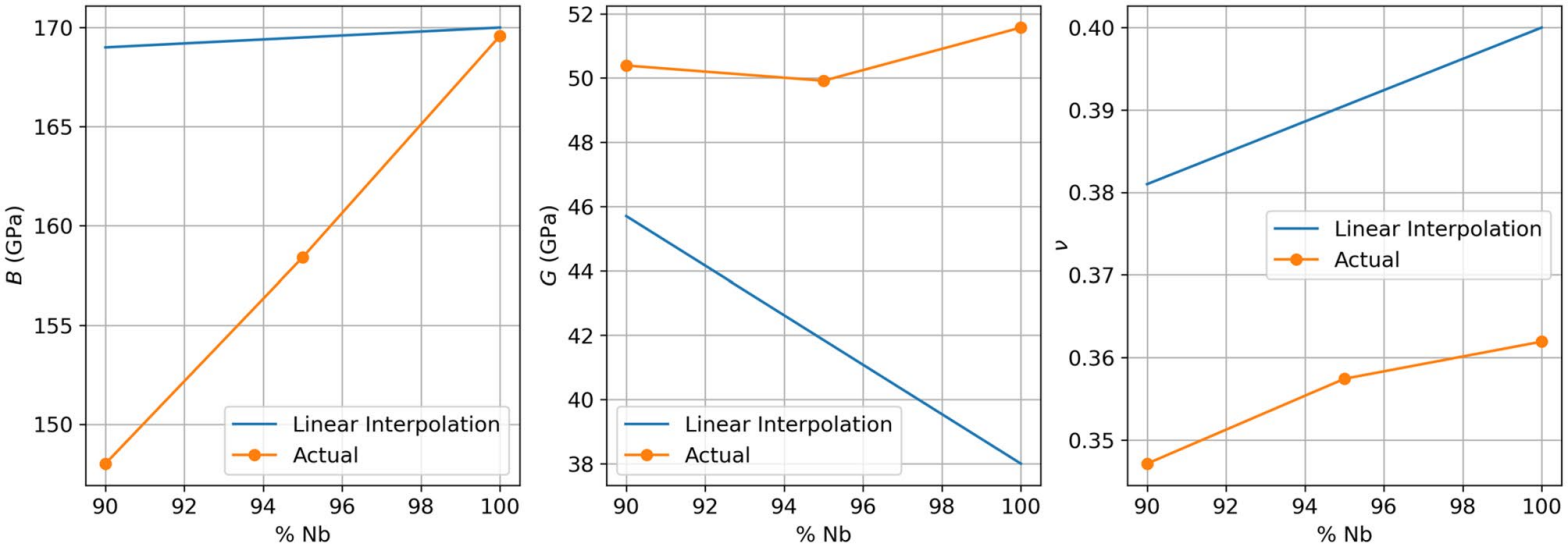
Comparison of measured (orange) strength and ductility as a function of solute concentration and calculated (blue) strength and ductility as predicted by Suzuki and Maresca and Curtin at room temperature. Bulk moduli, shear moduli, and Poisson’s ratio values were taken from Macmillan’s Chemical and Physical Data^55^.

### Thermal conductivity

**Figure 11:**
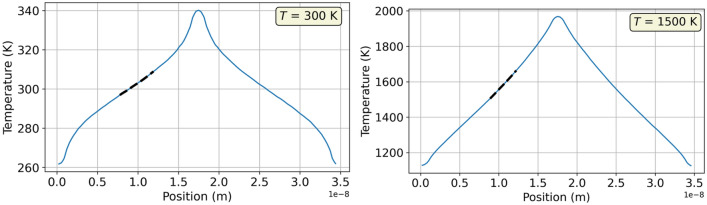
Temperature profiles in C15 NbCr$_{2}$.

Thermal conductivities for C15 NbCr$_{2}$ and three Nb-Cr alloys are summarized in Table 5. Two temperature profiles generated from the Müller-Plathe rNEMD method (Sect. 4.5) are also shown in Fig. 11. Thermal conductivity is relevant to studies involving materials exposed to extreme temperatures. For example, materials used as heat sinks intended to dissipate heat away from a certain area must have a high thermal conductivity to work effectively. In examining Table 5 and comparing the alloys, it can be seen that larger amounts of Cr are accompanied by a decrease in thermal conductivity. This is consistent with the rule of mixtures^56^ and with Jiang et al^42^, which reported an increase in thermal conductivity in W-Cr alloys with increasing amounts of Cr. A decrease in thermal conductivities with temperature for all materials can also be observed, which is reasonable and consistent with the literature^57^, and with the fact that anharmonic phonon scattering rates usually increase with temperature, causing the decrease of thermal conductivity. The decrease in thermal conductivities with increasing amount of Cr is also consistent with Vegard’s law. While the decrease is not linear, the smaller thermal conductivities suggest smaller equilibrium lattice parameters in the cells with increased amounts of Cr, acting to reduce the mean free path of phonons. Thermal conductivities are only reported for the alloys at two or three temperatures due to unphysical heat fluxes predicted at higher temperatures, likely a shortcoming of the employed interatomic potential and its inability to predict behavior in a system with a large temperature gradient. In the case of pure Nb, direct comparisons with literature^58^ reveal that all three values reported are approximately twice as large as those predicted by DFT calculations. We attribute this overestimate to the aforementioned deficiencies in our EAM potential. However, the variation of the thermal conductivity with temperature is consistent with our knowledge and the available literature.

**Table 5:** Summary of thermal conductivities in W/m-K.

**Material**	**300 K**	**600 K**	**900 K**	**1200 K**	**1500 K**
C15	9.27	5.37	3.83	3.06	2.52
90% Nb, 10% Cr	1.1681	0.8277	N/A	N/A	N/A
95% Nb, 5% Cr	2.27	2.20	1.66	N/A	N/A
Pure Nb	19.97	10.52	6.37	N/A	N/A

### Heat capacity

**Figure 12:**
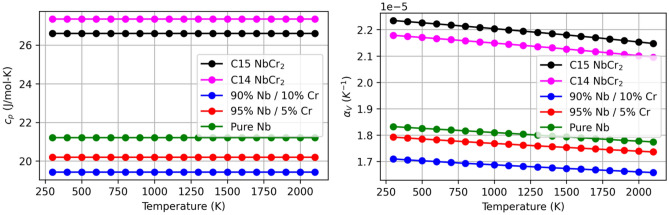
Heat capacities (left) and volumetric thermal expansion coefficients (right) in various Nb-Cr systems. In the alloys, $c_{P}$ and $\alpha _{v}$ are shown to decrease with Cr content.

Heat capacities in C14 and C15 NbCr$_{2}$ and three Nb-Cr alloys as a function of temperature are shown in Fig. 12. As heat capacity is a measure of the amount of energy necessary to produce a one temperature unit change in a single mole of a given material, heat capacity is a highly relevant property in materials proposed for high-temperature applications. In our findings, we note that the predicted heat capacity of approximately 21 J/mol-K for pure Nb matches well with the known value at ambient temperature of 24.16 J/mol-K^59^ and decreases with increasing amounts of Cr. As the heat capacity of Cr is lower than that of Nb^55^, this is qualitatively consistent with the rule of mixtures, which suggests that material properties can be interpolated based on the solute concentration^56^. Furthermore, it is consistent with Jiang et al^42^, which predicted a similar decrease in W-Cr alloys. Additionally, the Laves-phase structures demonstrate considerably higher heat capacity than the alloys, with the C14 structure having a slightly higher capacity than C15. This is consistent with the higher thermal stability observed in Laves-phase structures, as compared to alloys^1,3,4^. All heat capacities were computed by taking the derivative of enthalpy with respect to temperature.

8 $$\begin{aligned} c_P={\left( \frac{\partial H}{\partial T}\right) }_P \end{aligned}$$

### Volumetric thermal expansion coefficient

Volumetric thermal expansion coefficients describe the change in volume that a material exhibits as a result of temperature changes. This particular property arises often in the context of higher temperature applications, where researchers and engineers may be concerned about the mismatch in expansion coefficients between an oxide that grows on the skin of an aircraft and the underlying metal, for example. In the present work, thermal expansion coefficients for the five materials investigated are shown to decrease with temperature, consistent with the literature^24^. As a linear relationship between volume and temperature was predicted for all materials, a linear decrease in the thermal expansion coefficient was anticipated. The C15 and C14 structures are shown to have higher thermal expansion coefficients than all alloy compositions, with the C15 structure exhibiting slightly higher volume sensitivity to temperature than the C14 material. The average expansion coefficient we report of 2.2 x 10^−5^ K$^{-1}$ for C15 NbCr$_{2}$ is in excellent agreement with Samanta et al^24^ which reported an average value of 2.5 x

10^−5^ K$^{-1}$. Mirroring its effect on heat capacity, increased Cr content is shown to result in lower thermal expansion coefficients in Nb-rich alloys. This is consistent the rule of mixtures and with Jiang et al^42^, which reported a decrease in thermal expansion coefficients in W-Cr alloys with increasing amounts of Cr. Expansion coefficients were calculated via

9 $$\begin{aligned} {\alpha }_V=\frac{1}{V}{\left( \frac{\partial V}{\partial T}\right) }_P \end{aligned}$$

### Gibbs free energy calculation

**Figure 13:**
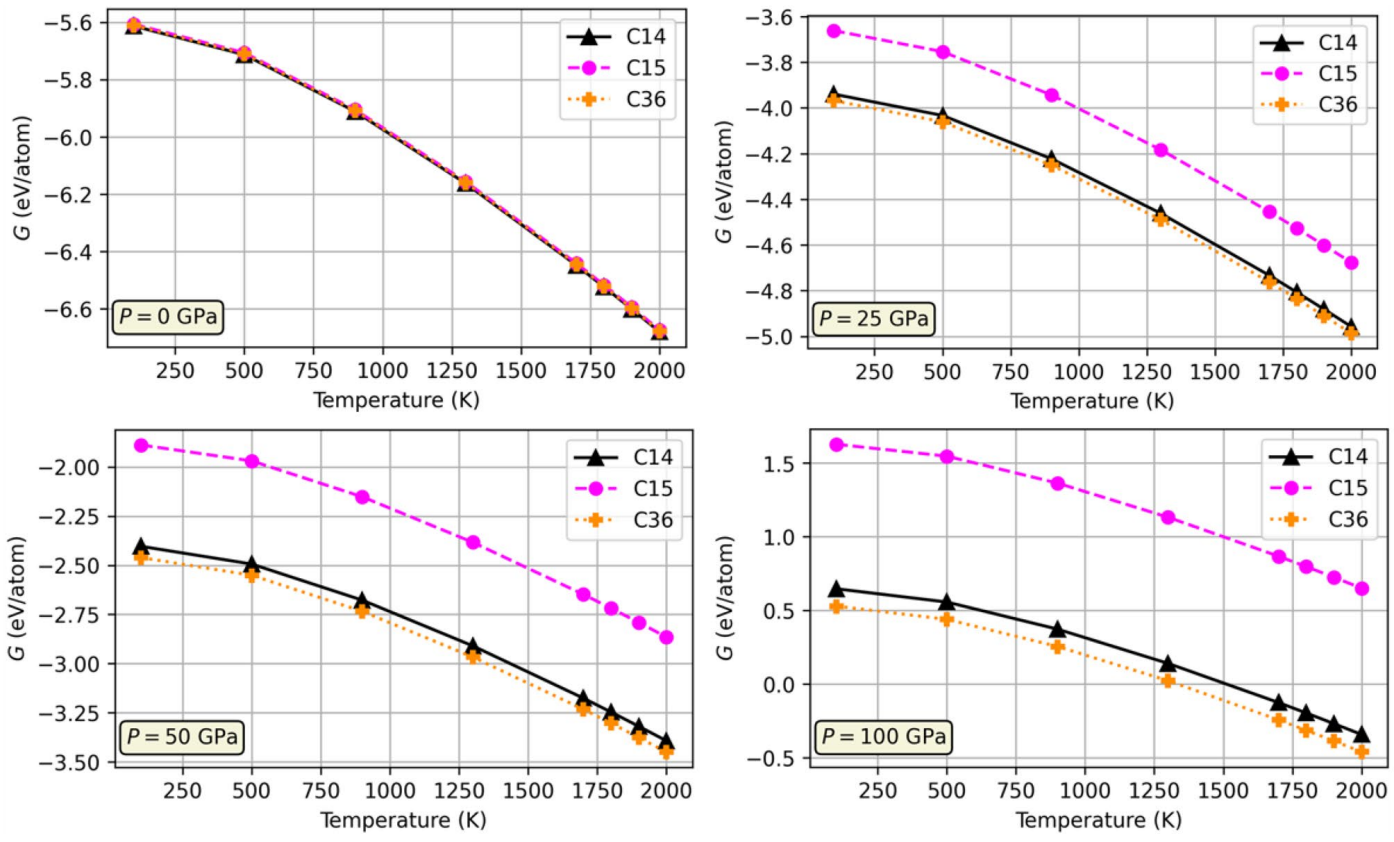
Gibbs free energies of C14, C36, and C15 NbCr$_{2}$ structures.

As discussed in the introduction, there appears to be some disagreement in the literature concerning the stability of the C14 and C15 NbCr$_{2}$ structures. In an attempt to contribute to the discussion on their stability, Gibbs free energies for all three NbCr$_{2}$ allotropes (C14, C15, and C36) were calculated over a range of temperatures and four different pressures. The specific methodology is outlined in Sect. 4.6. Figure 13 compares the free energies of each structure as a function of temperature and pressure. The C36 phase, which adopts a hexagonal crystal structure very similar to C14 and is experimentally observed at high pressures, is observed to be more stable (lower in energy) than both the C14 and C15 phases in all cases. Furthermore, the difference in free energies between the three structures are observed to increase with increasing pressure. These findings suggest that C36 is the most stable phase for all temperatures and pressures considered and becomes increasingly more stable as pressure increases.

## Discussion

By developing a basic interatomic interaction potential for Nb-Cr (which did not exist in the literature), we have attempted to gain insight into the effect of Cr solutes in Nb by characterizing thermomechanical performance as a function of Cr content. Additionally, this study reported the temperature dependence of thermal conductivity, heat capacity, and the volumetric thermal expansion coefficients for the same alloys and both the C14 and C15 Laves-phase NbCr$_{2}$ structures. An analysis of the thermodynamics of the C15, C14, and C36 NbCr$_{2}$ structures was also carried out by calculating Gibbs free energies for the three intermetallics over a range of temperatures and pressures.

Summarizing our results, we report an apparent effect of Cr content on the mechanical properties of the three investigated Nb-Cr alloys. Increased Cr content was found to reduce Young’s modulus, yield strength, ultimate tensile strength, and ductility, as well as accelerate the onset of plasticity in each material and increase crack growth rates. Young’s modulus, the bulk modulus, and Poisson’s ratio were all found to decrease with Cr content. Furthermore, elasticity in the alloys was found to satisfy the Born-Huang stability criteria and all alloys were predicted to exhibit bond stretching according to the Kleinman parameter. Additionally, in reviewing the concentration dependence of strengthening, we observed evidence of solid solution strengthening based on screw dislocations as outlined by Suzuki^52^ and Maresca and Curtin^53^. Nearly all behaviors were found to be consistent with those predicted in similar W-Mo alloys^51^. In terms of the effect on thermal properties, all investigated alloys exhibited reduced heat capacities, thermal expansion coefficients, and thermal conductivities with increased amounts of Cr. These predictions were found to be in qualitative agreement with behaviors predicted in W-Cr alloys^42^. Gibbs free energies calculated using the reverse non-equilibrium technique were observed to be lower for the C36 phase as compared to the C14 and C15 phases for all investigated temperatures and pressures, suggesting that C36 NbCr$_{2}$ is stable over a wide range of environments, inconsistent with the literature. However, the C36 phase tended to become more stable relative to the other two phases as pressure increased.

Both the diminished strength in Nb-Cr and the reduced thermal conductivity, heat capacity, and thermal expansion as a result of increased Cr content are vital relationships to keep in mind for the design of high-strength Nb-Cr materials and represent a step towards improving our understanding of compositionally complex refractory alloys. Structure-property relationships like these are frequently studied in the literature and may enhance our knowledge and enable improved physics-based future designs. Related to this work, Cr content could be tailored to reduce the cracking and corrosion that occurs as a result of mismatched thermal expansion coefficients between an underlying metal and an oxide that forms on its surface. Nb$_{2}$O$_{5}$ and Cr$_{2}$O$_{3}$, two oxides that could be expected to form on the surface of an Nb-Cr alloy, have ambient temperature thermal expansion coefficients of 3x10$^{-5}$ K$^{-1}$ and 3x10$^{-6}$ K$^{-1}$^60^, respectively. Therefore, our prediction of thermal expansion coefficients of 1.7x10$^{-5}$ K$^{-1}$ for the 10 at% Cr and 1.8x10$^{-5}$ K$^{-1}$ for pure Nb suggest that alloys with higher amounts of Cr are more suitable for materials experiencing Nb$_{2}$O$_{5}$ or Cr$_{2}$O$_{3}$ growth. Moreover, our findings on the Laves-phase materials support efforts examining NbCr$_{2}$-based alloys, which dominate the literature on the Nb-Cr system.

Of course, we emphasize that this work is only a first step in understanding these materials, as there are limitations related to the development of the employed interatomic potential. In particular, this potential was not trained on hexagonal phases (or any non-cubic structure, for that matter) and the Nb-Cr interactions were not explicitly trained on. Instead, a cross-term formulation introduced by Johnson^33^ was adopted. Despite these limitations, the computations yielded reasonable results and helped provide qualitative insight into the properties of Nb-Cr alloys and compounds, the effect of Cr content, and the stability and thermomechanical performance of the intermetallic Laves phases. Going forward, we advocate for additional training of the employed EAM potential, specifically focusing on hexagonal structures and accounting for the stacking fault energies and vacancy formation energies, and explicit training of the cross-term using intermetallics. Including this data into the fitting process would yield a potential which can more comprehensively predict behaviors in materials with complex crystal structures, such as C14 NbCr$_{2}$. Furthermore, explicitly training the pairwise interaction between Nb and Cr, rather than using a cross-term formula could result in a better representation of the Nb-Cr system.

## Methods

### General

Molecular dynamics (MD) simulations were carried out using the Large-scale Atomic Molecular Massively Parallel Simulator (LAMMPS) code^61^. Dislocation densities and phase transformation diagrams were generated using crystal analysis algorithms as implemented in OVITO^50^. The OVITO software was also used for general system visualization, as well. The ATOMSK code^62^ was used to generate all simulation cells. The average of two structures was used to capture different ensemble mechanisms for the Nb-Cr solid solution data points. The differential displacement map (Fig. 4) was created using the ATOMMAN dislocation analysis tool as implemented in Python and published by the National Institute of Standards and Technology (NIST)^63^.

### Interatomic potential & embedded atom model

The interatomic potential used in this work was developed according to the embedded-atom model (EAM) as outlined by Zhou and Wadley^64^. An explanation of the formulation is provided below. In Zhou and Wadley’s description, the interaction energy between two atoms is given by

10 $$\begin{aligned} E_{ne}=\frac{1}{2}\sum ^N_{i=1}{\sum ^{i_M}_{j=i_1}{\varphi _{ij}(r_{ij})}}+\sum ^N_{i=1}{F_i(\rho _i)} \end{aligned}$$

where $$\varphi$$$${}_{ij}$$(*r*$${}_{ij}$$) describes the pairwise interaction between atoms *i* and *j*, *F*$${}_{i}$$ represents the embedding energy, and $$\rho$$$${}_{i}$$ is the background electron density. The ‘*ne*’ subscript indicates a non-electrostatic interaction. A Morse-like function describes the pairwise interaction and is divided by a cutoff radius according to Equation 11.

11 $$\begin{aligned} \phi (r)=\frac{A\exp {\left[ -\alpha \left( \frac{r}{r_e}-1\right) \right] \ }}{1+{\left( \frac{r}{r_e}-\kappa \right) }^{20}}-\frac{B{exp \left[ -\beta \left( \frac{r}{r_e}-1\right) \right] \ }}{1+{\left( \frac{r}{r_e}-\lambda \right) }^{20}} \end{aligned}$$

The energy required to embed an atom into a crystal lattice with a background electron density is given by Equation 12. $\rho _e$ represents the equilibrium electron density for a ground-state crystal.

12 $$\begin{aligned} F(\rho _i)=\left\{ \begin{array}{c} \sum ^3_{i=0}{F_{n_i}{\left( \frac{\rho _i}{\rho _n}-1\right) }^i}\mathrm {,\ \ \ \ \ \ \ \ \ \ \ \ \ \ \ \ \ \ \ \ }\rho \mathrm {<}\rho _n,\ \rho _n=0.85\rho _e \\ \sum ^3_{i=0}{F^-_i{\left( \frac{\rho _i}{\rho _e}-1\right) }^i}\mathrm {,\ \ \ \ \ \ \ \ \ \ \ \ \ \ \ \ \ \ \ \ }\rho _n\le \rho<\rho _e \\ \sum ^3_{i=0}{F^+_i{\left( \frac{\rho _i}{\rho _e}-1\right) }^i}\mathrm {,\ \ \ \ \ \ \ \ \ \ \ \ \ \ \ \ \ \ \ \ }\rho _e\le \rho <\rho _o,\ \rho _n=1.15\rho _e \\ F_e\left[ 1-{{ln \left( \frac{\rho }{\rho _s}\right) \ }}^\eta \right] {\left( \frac{\rho }{\rho _s}\right) }^\eta \mathrm {,\ \ \ \ \ \ \ \ }\rho _0\le \rho \end{array} \right. \end{aligned}$$

The electron density, $\rho _i$, is computed as a sum of atomic electron densities *f*${}_{i}$ from neighboring atoms *j* around atom *i.* While this calculation is performed “on-the-fly” in the MD code, we include the equation here for reference.

13 $$\begin{aligned} \rho _i=\sum ^N_{i=1}{f_i(r_{ij})} \end{aligned}$$

14 $$\begin{aligned} f_i(r_{ij})=\frac{f_e{\exp \left[ -\beta \left( \frac{r}{r_e}-1\right) \right] \ }}{1+{\left( \frac{r}{r_e}-\lambda \right) }^n} \end{aligned}$$

There are 21 parameters per atom type. No additional parameters were included for the cross-term interactions between metals *a* and *b*. These were modeled via Johnson’s cross term formulation^33^, shown in Equation 15. Plots showing the pairwise interaction, embedding energy, and the electron density (equations 11, 12, and 13) are shown in Figure 3 of the Supplementary Information.

15 $$\begin{aligned} {\phi }_{ab\left( r\right) =}\frac{1}{2}\left( \frac{f_b\left( r\right) }{f_a\left( r\right) }{\phi }_{aa}(r)+\frac{f_a(r)}{f_b(r)}{\phi }_{bb}\right) \end{aligned}$$

All parameters were fitted to DFT-calculated lattice constants, cohesive energies, equations of state, and elastic coefficients for bcc and fcc Nb and Cr. No Nb-Cr compounds or other mixed structures were used in the training process. Optimization was accomplished using a trust region reflective algorithm as implemented in Python 3.10. In simple terms, this method involves approximating the objective function by a second-order Taylor series polynomial within a neighborhood (‘trust region’) around the point of interest. The solver then computes a trial step by minimizing the proxy function over the trust region. If the value of the objective function at the calculated trial step is smaller than the objective function at the original point, the solution vector is updated. If it is larger, the trust region is made smaller and a trial step is computed again. This process is repeated until a specified error tolerance is achieved or the solver has executed the maximum number of function evaluations. In this work, function, gradient, and solution vector tolerances were set to 3x10$^{-16}$. The number of function evaluations was capped at 100*n*, where *n* is the number of independent variables.

#### Density functional theory calculations

Density Functional Theory (DFT) calculations were carried out using the projector augmented plane wave (PAW) method^65^ for the electron-ion interaction as implemented in the Vienna Ab initio Simulation Package (VASP)^66,67^. The generalized gradient approximation (GGA)^68^ was used for the exchange correlation functionals as parametrized by Perdew-Burke and Ernzerhof (PBE)^69^. The employed projector augmented wave pseudopotentials treated the following electrons as valence states: Nb (4p6 5s1 4d4), and Cr (3p6 4s1 3d5). The Kohn-Sham orbitals were expanded using a plane wave basis set with a cutoff of 600 eV. Brillouin zone integrations were performed using a Monkhorst-Pack grid^70^ and a Methfessel-Paxton smearing^71^ width of 0.1 eV was employed in all simulations. The k-point mesh was converged for each structure. Structural parameters were optimized by simultaneously minimizing all atomic forces via a conjugate gradient algorithm. The self-consistent calculations were converged to an accuracy of 10-7 eV and the atomic relaxation steps were allowed to continue until the energy of the system satisfied a tolerance threshold of 10-4 eV between successive ionic relaxation steps. The elastic constants were computed by introducing small perturbations of 0.01 Å and calculating the second derivatives of the energy with respect to these distortions using central differencing as implemented in VASP.

### Crack propagation method

Crack propagation studies were carried out using an input script adopted from the LAMMPS binary^72^. In this implementation, the velocities of the top five layers of atoms were set to 0.15 Å/ps, while the bottom five layers were fixed in place. The velocities of the middle atoms were linearly interpolated according to their position relative to the top and bottom of the simulation cell. In other words, atoms near the top of the simulation cell were assigned velocities close to 0.15 Å/ps, and atoms near the bottom were assigned velocities close to zero. Following these assignments, a two nanosecond production run in the NVE ensemble was carried out and snapshots of the trajectory were recorded every 1000 time steps. To extract the crack tip position, the resulting dump file was split in half in the *x* direction and the simulation cell was binned along the *y* dimension. After identifying the *y* position of the crack tip by finding the bin with the fewest number of atoms, the crack tip location in the *x* direction was identified by taking the minimum of the *x* coordinates of all atoms in the identified bin. 40 bins was deemed an adequate number to properly identify the position of the crack tip. A cubic simulation cell of 128,000 atoms with dimensions of 13.2 nm in each direction was adopted. The *x* direction was oriented along [100], the *y* along [010] and the *z* along [001]. In this method, an opening (mode I) crack is permitted to form during the simulation. No cracks are inserted prior to applying the load.

### Equilibrium method for calculating elastic coefficients

Elastic coefficients were calculated according to the explicit deformation method outlined in Clavier et al^73^. In this equilibrium method, elementary deformations are imposed on the system and C$_{ij}$ is taken as the proportionality coefficient between the stress component $\sigma _{i}$ and the strain element $\epsilon _{j}$. Elongations and compressions of the supercell are used to determine the matrix elements along the diagonal and shear strains, wherein the angle between box vectors is modified by some amount, are used to extract the off-diagonal elements.

### Reverse non-equilibrium method (rNEMD) for calculating thermal conductivity

Thermal conductivities were calculated using the reverse non-equilibrium method (rNEMD) introduced by Müller-Plathe^74^. In this method, a temperature gradient is generated and Fourier’s law (Equation 16) is used to extract the thermal conductivity. The technique involves creating a supercell of atoms that is in one dimension (typically the ’*z*’ dimension) “infinitely” long compared to the other two (i.e. $L_{z}>> L_{yy} = L_{x}$). The supercell is then binned along this dimension and two bins are designated the “hot” and “cold” bins. The temperature gradient is generated across the supercell by swapping the velocity vectors of the “hottest” and “coldest” atoms in each bin. In doing so, an energy transfer from the cold bin to the hot bin occurs and is eventually balanced by a heat flux in the opposite direction. At this point, a well-defined temperature profile is realized and the total kinetic energy transferred in performing the swaps is recorded. From here, the thermal conductivity, $\kappa$, is calculated using Equation 16.

16 $$\begin{aligned} J_z= & {} -\kappa \frac{dT}{dz} \end{aligned}$$

17 $$\begin{aligned} \kappa= & {} \frac{{KE}_{trans}}{2L_xL_yt\frac{dT}{dz}} \end{aligned}$$

In Equation 16, ${KE}_{trans}$ represents the cumulative kinetic energy transferred, $\frac{dT}{dz}$ is given by the slope of the linear portion of the temperature profile, and $L_x$ and $L_y$ represent the cell widths in the *x* and *y* directions, respectively. *t* is the total time over which the kinetic energy was transferred. The “2” in the denominator accounts for heat flux traveling in both directions, which effectively doubles the cross sectional area of the supercell. In this work, to ensure adequate convergence in the thermal conductivity estimates as explored in El-Genk et al^57^, we employed a supercell of 10x10x500 UC.

### Non-equilibrium method for calculating gibbs free energies

Gibbs free energies were computed using a non-equilibrium method described in Freitas et al^75^. In this technique, the system is evolved between a reference state and a state of interest over a switching time, $t_{s}$. The irreversible work done in moving between the states is then calculated in both in the forward ($i\rightarrow f$) and backward ($f\rightarrow i$) directions according to Equation 18, where $\lambda$ is a coupling parameter that determines the state of the system and *H* is the Hamiltonian as described in Equation 18.

18 $$\begin{aligned} W^{irr}_{i\rightarrow f}= & {} \int ^{t_s}_0{dt\frac{d\lambda }{dt}}{\left( \frac{\partial H}{\partial \lambda }\right) }_{{\Gamma }_{(t)}} \end{aligned}$$

19 $$\begin{aligned} H\left( \lambda \right)= & {} \lambda H_f+(1-\lambda )H_i \end{aligned}$$

In Equation 18, $H_{i}$ and $H_{f}$ represent the initial (reference) and final states, respectively. The final state is defined by the employed interatomic potential. The reference state is chosen cleverly as the Einstein crystal, as its Helmholtz free energy, $F_{E}$, can be described analytically. The Helmholtz free energy of the state of interest is computed via Equation 20. A transformation to the Gibbs free energy is then made via Equation 20.

20 $$\begin{aligned} F\left( N,V,T\right)= & {} F_E\left( N,V,T\right) +\frac{1}{2}\left[ \left\langle W^{irr}_{1\rightarrow 2}\right\rangle -\left\langle W^{irr}_{2\rightarrow 1}\right\rangle \right] \end{aligned}$$

21 $$\begin{aligned} G(N,P,T)= & {} \underset{V}{\min }\left[ F(N,V,T)+PV \right] \end{aligned}$$

**Figure 14:**
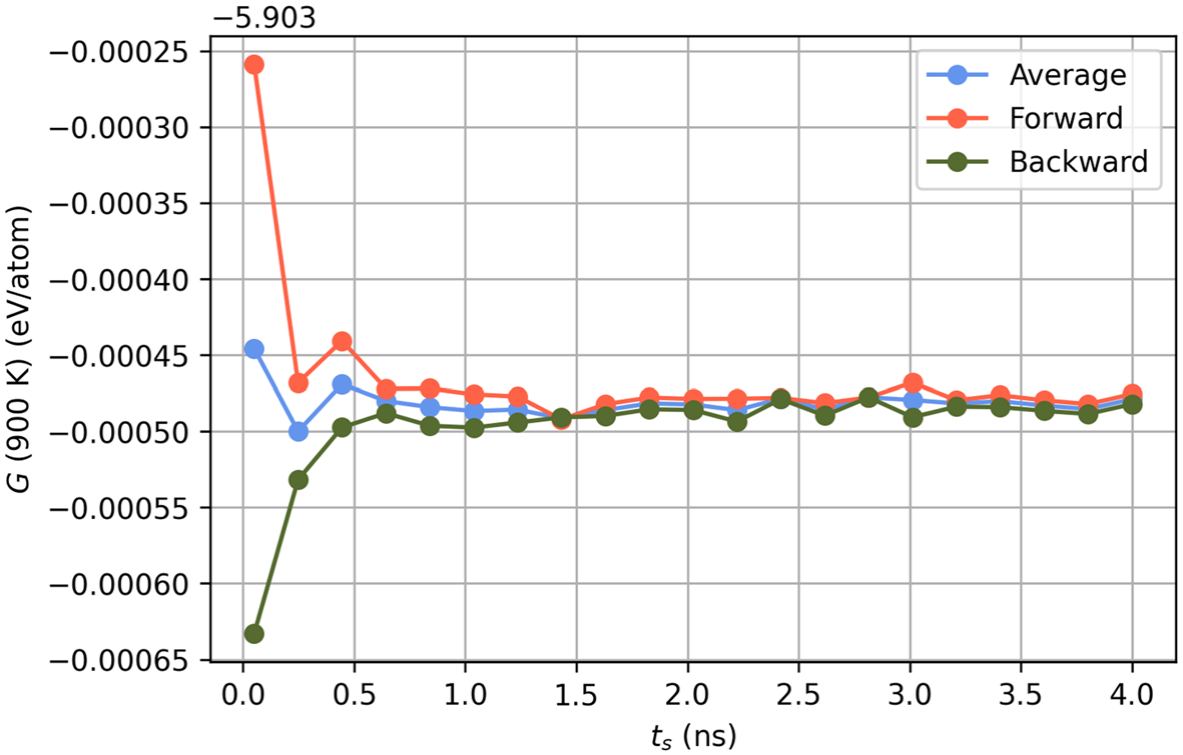
Gibbs free energy as a function of switching time for C15 NbCr$_{2}$ at 900 K.

In this work, a switching time of 8 ns was used. This was found to be more than adequate, as convergence in the Gibbs free energies for the C15 NbCr$_{2}$ structure at 900 K was achieved with a switching time of approximately 1.5 ns (Fig. 14).

### Supplementary Information

Supplementary Figures.

## Data Availability

The interatomic potential used in this work and the corresponding DFT data is available at https://github.com/lheaton4/NbCr_EAM_Potential_and_DFT_data. All other scripts and data used in this work may be made available upon reasonable request by contacting the corresponding author, Adib Samin.
