# Supplementary material for: A first step towards understanding thermomechanical behavior of the Nb-Cr system through interatomic potential development and molecular dynamics simulations
Source: Sci Rep. 2024 Jun 22;14:14408. doi: 10.1038/s41598-024-64920-w (PMC11193778; doi:10.1038/s41598-024-64920-w)
Supplement: Supplementary file 1 — Supplementary Figures. [file 41598_2024_64920_MOESM1_ESM.docx]

Supplementary Information

*A First Step Towards Understanding Thermomechanical Behavior of NbCr Solid Solutions and the Stability of NbCr_2_ Laves Phases Through Interatomic Potential Development and Molecular Dynamics Simulations*

*Lucas A. Heaton & Adib J. Samin*


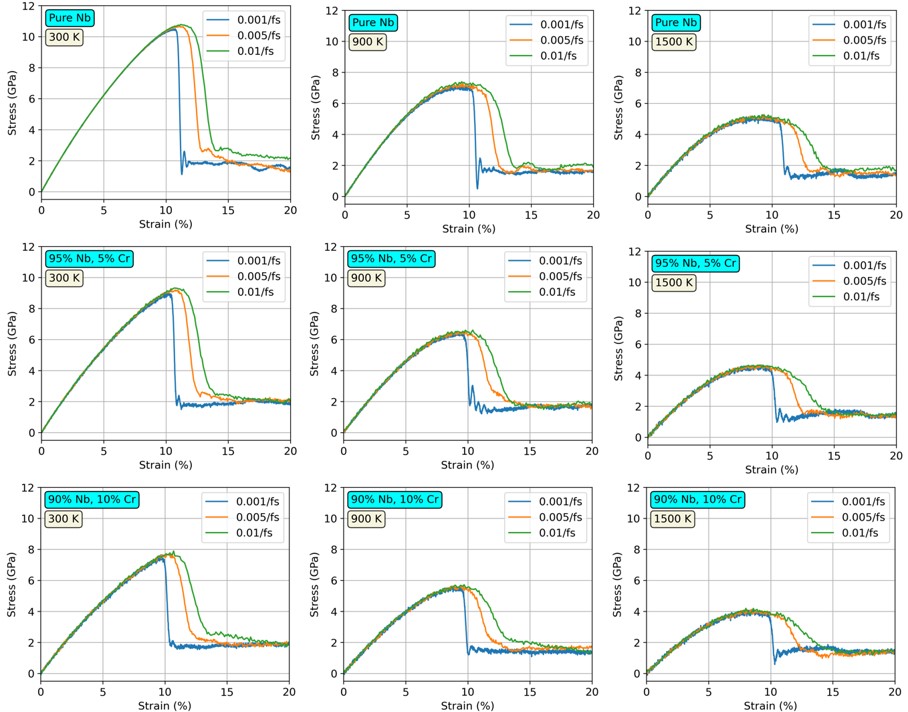


Figure 1: Stress-strain curves in Nb solid solutions as a function of temperature and strain rate.


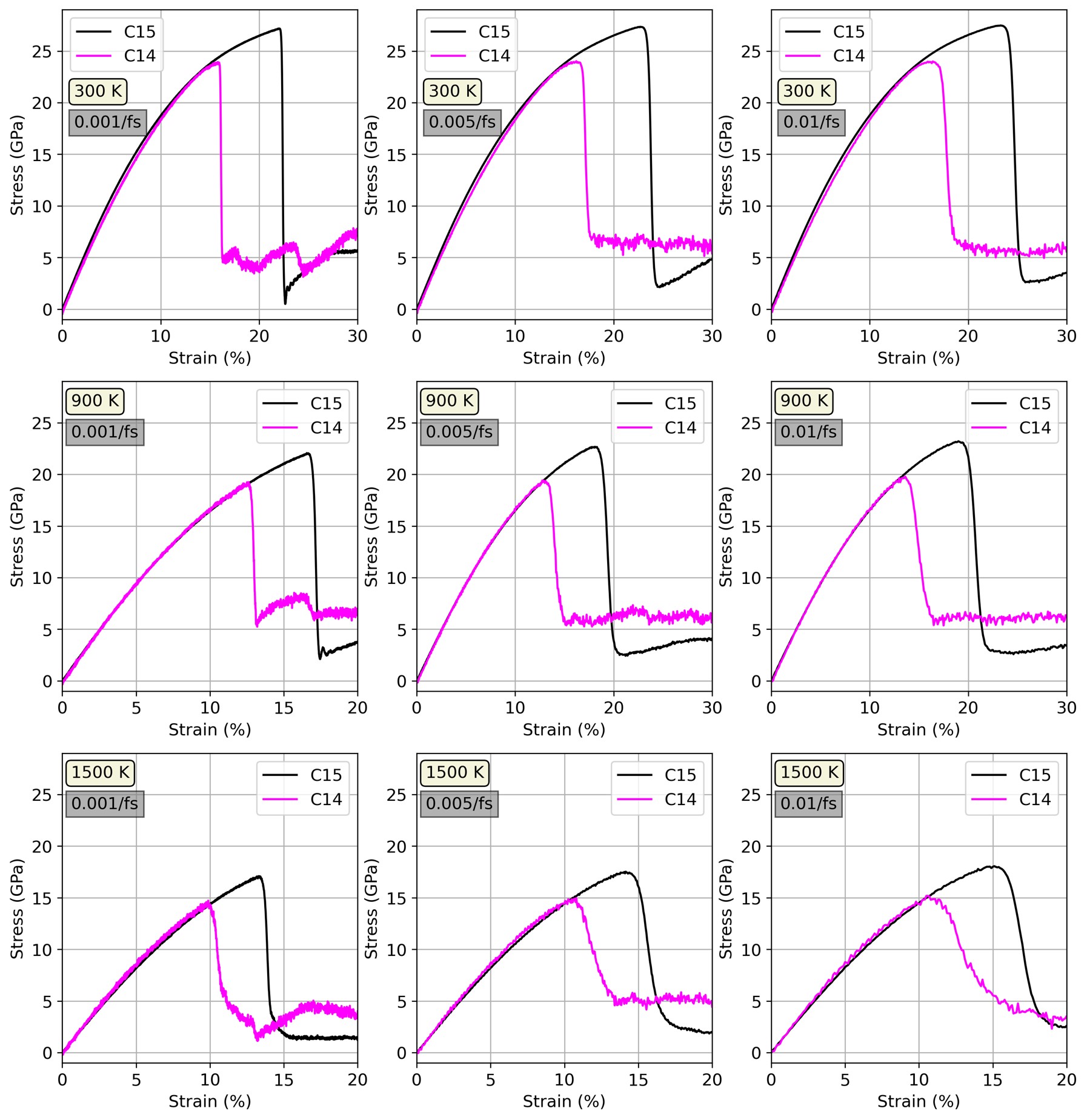


Figure 2: Stress-strain curves in Laves NbCr_2_.


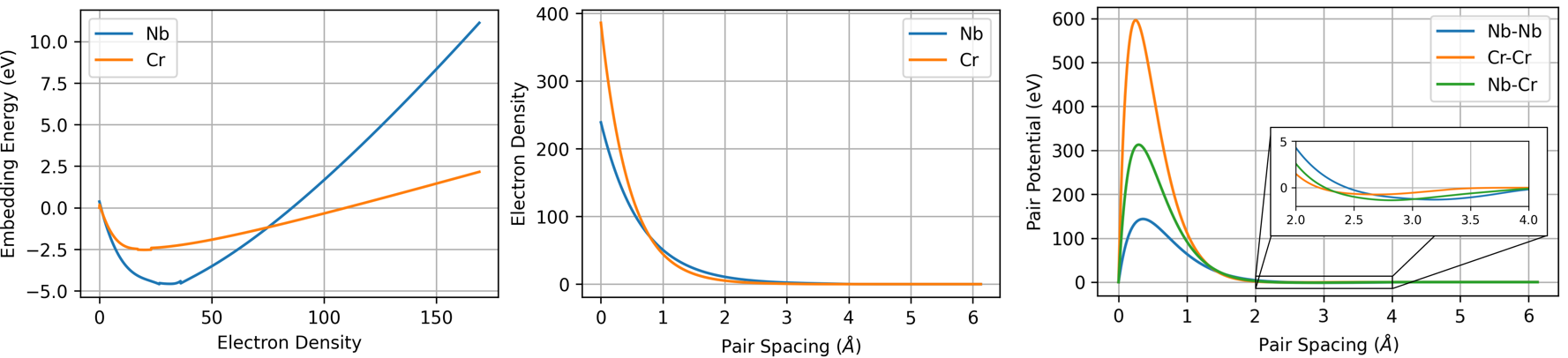


Figure 3: Embedding energy, electron density, and pairwise potential of EAM potential. An inset showing the attractive part of the potential can be seen at right.
